# Investing in global health security: Estimating cost requirements for country-level capacity building

**DOI:** 10.1371/journal.pgph.0000880

**Published:** 2022-12-05

**Authors:** Stephanie Eaneff, Ellie Graeden, Amanda McClelland, Rebecca Katz

**Affiliations:** 1 Georgetown University Center for Health Science and Security, Washington, D.C., United States of America; 2 Resolve to Save Lives, Washington, D.C., United States of America; PLOS: Public Library of Science, UNITED STATES

## Abstract

The COVID-19 pandemic has highlighted critical gaps in global capacity to prevent, detect, and respond to infectious diseases. To effectively allocate investments that address these gaps, it is first necessary to quantify the extent of the need, evaluate the types of resources and activities that require additional support, and engage the global community in ongoing assessment, planning, and implementation. Which investments are needed, where, to strengthen health security? This work aims to estimate costs to strengthen country-level health security, globally and identify associated cost drivers. The cost of building public health capacity is estimated based on investments needed, per country, to progress towards the benchmarks identified by the World Health Organization’s Joint External Evaluation (JEE). For each country, costs are estimated to progress to a score of “demonstrated capacity” (4) across indicators. Over five years, an estimated US$124 billion is needed to reach “demonstrated capacity” on each indicator of the JEE for each of the 196 States Parties to the International Health Regulations (IHR). Personnel costs, including skilled health, public health, and animal health workers, are the single most influential cost driver, comprising 66% of total costs. These findings, and the data generated by this effort, provide cost estimates to inform ongoing health security financing discussions at the global level. The results highlight the significant need for sustainable financing mechanisms for both workforce development and ongoing support for the health and public health workforce.

## Introduction

The ongoing COVID-19 pandemic has highlighted clear gaps in global capacity to prevent, detect, and effectively respond to emerging and existing infectious disease threats. Global leaders are engaged in robust discussions to create more sustainable, substantial financing for global health security. However, to effectively allocate investments that address existing gaps in health security, it is necessary to quantify the extent of the need, evaluate the types of resources and activities that require additional support, and engage the global community in ongoing assessment, improved planning, and accelerated implementation. How much will it cost to address ongoing and future outbreaks before they become pandemics? What investments are needed, where, to strengthen national public health systems?

Despite ongoing global dialogue on the need for additional investment in health security, limited information is available to estimate the magnitude of these costs or to articulate specific investment requirements. What estimates do exist vary both in terms of dollar amounts and in their assumptions regarding what is and is not included in cost estimates [1–3]. Limited alignment between existing work makes “apples-to-apples” comparisons between cost estimates difficult; moreover, it is challenging to identify the assumptions and underlying methodological differences that contribute to variability in estimated costs.

Over the past ten years, our team has developed and maintained the IHR Costing Tool, an online resource to estimate the costs of national and subnational public health capacity building. Tool development was informed by case studies reviewing IHR implementation in 14 countries spanning multiple regions. The tool uses an action-based costing approach to estimate the cost to progress towards the benchmarks articulated by the Joint External Evaluation (JEE) over a five-year period [4]. As such, the tool aligns to the expectations set by the JEE for public health capacity building under the International Health Regulations (IHR 2005) across 19 technical areas including national legislation, policy and financing, immunization, national laboratory system, surveillance, reporting, and risk communication [4]. The tool has been validated against other costing frameworks and costed national action plans [5], and a version of the tool and the associated underlying datasets is available in both English and French at ghscosting.org.

Building upon prior research, the work described here aims to identify gaps in country-level health security, globally, estimate the cost to fill those gaps, and identify cost drivers that contribute substantially to overall investment needs. We used the IHR costing tool and underlying data to estimate national and subnational costs among all 196 States Parties to the IHR. Results are aligned with other independent cost estimates to enable global dialogue regarding costing and subsequent financing requirements to build country-level capacity to prevent, detect, and respond to infectious diseases. As such, results are intended to be used and interpreted as high-level order of magnitude estimates of the costs to build country-level capacity, globally, not as estimates of individual country-level resource needs.

Sustainable and equitable investment in global health security must be informed by a robust understanding of current capacity and gaps, ongoing engagement with national and subnational stakeholders who best understand conditions on the ground, and an understanding of existing standards and benchmarks for measuring health security. Such information can be used to target additional investments in health security and health systems strengthening where they are needed most. Quantitative approaches for assessing gaps in capacity can also help to articulate where additional policy guidance is needed–particularly for areas of health security where substantial investment is needed but where existing benchmarks may be vague or insufficient.

## Methods

### Measure country-level capacity across indicators

The financial cost of building country-level public health capacity, globally, under the International Health Regulations (IHR) was estimated based on anticipated investments necessary for each of the 196 States Parties to the IHR to progress towards the criteria identified by the Joint External Evaluation (JEE) [6]. Capacity for each State was measured based on State Parties Self-Assessment Annual Reporting Tool (SPAR) reports [7]. For States with publicly available SPAR reports, the most recently reported SPAR report or JEE mission report as of March 2021 was used to estimate current capacity. As of this time, all but 4 States Parties (Barbados, Brunei Darussalam, Bolivia, and Grenada) had publicly available e-SPAR assessments. Brunei Darussalam had a publicly available JEE mission report from 2019; for the remaining 3 countries, capacity was estimated based on the mean of reported scores by countries within the WHO region.

The IHR Costing Tool relies primarily on indicator-level ratings that align with the first edition JEE. The JEE measures health security capacity on a five-point scale ranging from “no capacity” (indicating that a capacity is not in place) to “sustainable capacity” (indicating that all attributes of a capacity are functional and sustainable). A score of “demonstrated capacity” corresponds to capacity that is in place but not yet fully functional and/or sustainable [6]. To align SPAR and JEE scores, SPAR scores were scaled from 1–5 based on guidance from WHO [8], and indicators were cross-walked between measures based on an extension of previously documented methods [9]. As the SPAR does not specifically consider immunization, items P.7.1 and P.7.2 of the JEE were assessed based on published measles vaccination data available from the WHO Global Health Observatory [10]. Additional details on the alignment of SPAR data, data from WHO, and the JEE is included in the Supplemental Appendix (S1 Table).

### Estimate costs

For each country, financial costs were estimated to progress to a score of “demonstrated capacity”(4) on all indicators of the first edition JEE over a five year period, based on the investments needed to complete the actions specified in the JEE across 19 technical areas and 48 indicators that span these technical areas. For example, the technical area “surveillance” includes an indicator assessing “interoperable, interconnected, electronic real-time reporting system(s)”. To progress from a score of “developed” (3) to “demonstrated” (4) capacity in this indicator, countries must establish and maintain intermediate-level surveillance units, with associated costs including salary support for surveillance staff and IT officers, and broadband internet access for unit offices. For each country, costs for each action (e.g., establish and maintain intermediate-level surveillance units) are determined based on standardized base costs (e.g., salary per staff member, cost of broadband internet), and scaled based on multipliers related to population size and/or level of administrative organization (e.g., number of intermediate areas).). Analysis focused strictly on country-level investment requirements and did not take into account activities related to global public good (e.g., R&D, global analysis and coordination, etc). Additional details on the underlying costing methodological have been published previously and described in-depth [4].

All base costs were assumed to be consistent throughout the five-year period for which costs were estimated, with no adjustments for forecasted inflation or potential price fluctuations. Costs for durable goods were assumed to be consistent globally, as were standard costs for international public health consultants. Costs associated with staff and healthcare worker compensation were adjusted at the regional level based on published guidance on salaries from WHO CHOICE [11], and assumed an additional 60% overhead and associated technology costs for each salaried worker. The size of the current healthcare workforce was estimated, per country, based on data from the WHO Global Health Observatory [12], WHO Country Cooperation Strategies documents [13,14], and the World Bank [15,16]. For the purpose of analysis, skilled healthcare workers were considered to include physicians, nursing and midwifery personnel [12]. Country populations and income groups were assessed based on estimates provided by the World Bank for 2020.

Select cost estimates for the existing IHR costing tool were updated based on learnings from the COVID-19 pandemic. This included the addition of line-item costed activities for the procurement of additional diagnostic tests [17], additional risk communication resources and personnel time to combat disinformation and misinformation and build community trust, and a reduction in anticipated travel costs and meeting expenses due to an increase in virtual meetings. Analysis also took into account costs associated with growing the skilled healthcare workforce to meet requirements under the IHR. Research and policy-based estimates of skilled health worker density requirements vary, ranging from 16·5 to 44·5 skilled health workers per 10,000 population [18–20]. This analysis relies on an estimate at the low end of this range, aiming to identify a minimum number of skilled healthcare workers necessary to perform the basic activities articulated by the JEE. For countries reporting fewer than 16·5 skilled health workers per 10,000 population, the recurring cost of additional healthcare personnel salaries was incorporated into cost estimates for the indicators D.2.1, P.3.2, and R.2.4, which are anticipated to rely heavily on public health workforce per capita. For each of these indicators, additional personnel were costed only if the country had a score below “demonstrated capacity” (4) on the specified indicator. Skilled healthcare workforce estimates assume that 25% of additional required healthcare workforce would be hired in year 1 to help with planning and onboarding of additional staff, with an additional 25% hired in years 3, 4, and 5.

## Results

Over five years, we estimate a cost of $124 billion for country-level capacity, globally. These costs of approximately $3·20 per person per year reflect the investments required for all States Parties to the IHR to progress to a score of “demonstrated capacity” on each indicator of the Joint External Evaluation. Developing capacity in low and lower-middle income countries will require approximately 79% of 5-year, total costs, with an additional 21% of total costs needed in upper-middle income and high-income countries. Per capita investment requirements are highest for low-income countries, with an estimated investment requirement of $66·84 per capita needed over five years, or $13·37 per person per year. In all, we estimate investment needs of $15·98 per capita needed over five years, or approximately $3·20 per person per year (Table 1).

**Table 1:** Overall and per capita costs by World Bank income group. Per capita costs reflect the costs of investments over a period of five years. All costs reported in approximate 2021 USD, and rounded to the nearest hundred million, as such, numbers reported may not sum precisely to total.

World Bank Income	Total per capita cost (over 5 years)	Total cost (5 years)
Low income	$66·84	$45·9 billion
Lower middle income	$17·79	$52·5 billion
Upper middle & high income	$6·19	$25·5 billion
**Total**	**$15·98**	**$123**·**8 billion**

Estimated costs varied by country and by region, depending on both population size and average SPAR score within each country and region. States Parties in the WHO Regional Office for Africa (AFRO) report, on average, the lowest SPAR scores across indicators (average of 49 vs. global average of 65), and, as such, have the highest estimate of required five-year cost ($60·7 billion). In contrast, other regions with smaller total population sizes (e.g., Eastern Mediterranean) or higher reported capacity ratings (e.g., Europe, Americas) have lower relative costs required over five years (Table 2).

**Table 2:** Costs by World Health Organization Regional Office. All costs are reported in approximate 2021 USD, and rounded to the nearest hundred million, as such, numbers reported may not sum precisely to total. Regional average (mean) SPAR data based on 2020 SPAR summary data reported by WHO. Per capita costs calculated based on total cost divided by estimated country population.

WHO Region	Average SPAR score	Total cost (5 years)	Per capita cost (over 5 years), by pillar			
			Prevent	Detect	Respond	Other
Regional Office for Africa	49	$60·7 billion	$10·04	$25·10	$18·25	$0·85
Regional office for the Americas	72	$8·0 billion	$2·39	$2·97	$1·95	$0·50
Regional Office for Southeast-Asia	63	$9·9 billion	$1·14	$0·80	$2·87	$0·08
Regional Office for Europe	75	$13·6 billion	$2·92	$8·48	$2·42	$0·80
Regional Office for the Eastern Mediterranean	67	$20·7 billion	$7·12	$10·19	$10·73	$0·45
Regional Office for the Western Pacific	70	$11·0 billion	$1·52	$2·22	$1·62	$0·32
**Total**	**65**	**$123**·**8 billion**	**$3**·**46**	**$6**·**76**	**$5**·**34**	**$0**·**43**

### Selected cost drivers

**Fig 1:**
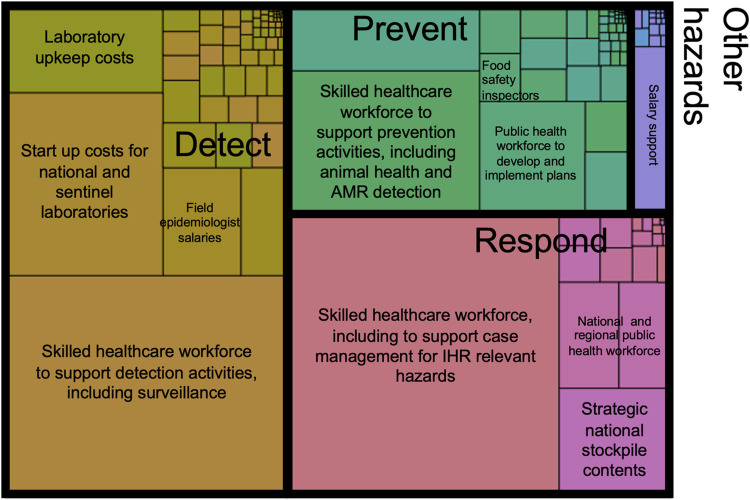
Distribution of 5-year costs, by pillar. Each cell corresponds to a costed line item and is scaled by cost and colored by pillar (e.g., prevent, detect, respond).

Overall, we find that activities related to detection and response are particularly high cost. Over a period of 5 years, we estimate costs of $52·4 billion (42% of total costs) for detection, $41·4 billion (33% of total costs) for response, $26·8 billion for prevention (22% of total costs), and $3·3 billion for other IHR-related hazards (3% of total costs). As indicated in prior research,^5^ primary cost drivers, across all pillars, include personnel costs, the costs of materials, especially those scaled at the population or facility level (e.g., stockpile kits per capita, laboratory facilities per intermediate area, or AMR training at hospitals) (Fig 1). Of note, while costs are discussed per indicator and pillar of the JEE based on the methodological approach described above, investments in health system strengthening are foundationally interconnected, such that investments in laboratory capacity, workforce development, and surveillance activities, among others, are beneficial across all domains of health security.

Personnel costs comprise 66% ($81·9/$123·8 billion) of total five-year costs and contribute substantially to overall costs of prevention, detection, and response. These workforce costs included both the estimated costs of additional skilled health workers (physicians, nurses, and midwives), as well as the costs of developing and maintaining an operational public health workforce, including laboratory workers, animal health workers, and support staff. Additional cost drivers included laboratory start-up at the intermediate and national levels, the cost of building and maintaining a strategic national stockpile (SNS) and the establishment of national and subnational facilities, including at designated points of entry (Table 3).

**Table 3:** Select cost drivers of total 5-year costs. All costs reported in approximate 2021 USD and rounded to the nearest tenth of a billion.

Pillar	Description of activity	Total cost estimate (5 years)
Prevent	Personnel costs for skilled healthcare workers	$10·5 billion
	Personnel costs for national animal health workers	$2·7 billion
Detect	Personnel costs for skilled health workers	$23·7 billion
	Start-up costs for national laboratory facilities	$10·8 billion
	Upkeep for national laboratory facilities	$5·1 billion
	Personnel costs for trained field epidemiologists	$3·2 billion
Respond	Personnel costs for skilled health workers	$29·4 billion
	Costs to stock and maintain a strategic national stockpile	$4·7 billion
Other	Establish and staff diagnostic facilities at designated points of entry	$2·3 billion

### Alignment with independent cost estimates

These investment estimates are consistent with the findings of independent analyses conducted by WHO and McKinsey and Company who, when considering the costs of country-level capacity building over a period of 5 years, estimate costs of $107·2 billion and $106-$204 billion, respectively [1,3] (Table 4). Estimates from the three sources align on several key findings, including the need for early upfront investments, and substantial additional investment requirements for personnel, surveillance capacity, and laboratory capacity. Where costs differ, it is largely due to differences in assumptions and scope. Costs increase with increased scope, and greater cost estimates from McKinsey are driven in part by the inclusion of a broader range of investments and activities including drug and vaccine R&D, manufacturing, and other investments in global public good. Similarly, the $95·6 B estimate from WHO accounts for only investment requirements in low-and-middle income countries and thus takes into account a smaller share of total need and of global population.

**Table 4:** Cost estimates for country-level pandemic preparedness across sources, reported in billions of 2021 USD. McKinsey and IHR costing estimates are rounded to the nearest billion. Note that these results do not include estimates from WHO or McKinsey for capacity building at the regional or global level in addition to the country level; McKinsey findings estimate that "73% percent [of total costs] would take place at the country level"[3].

Source	*Cost estimates by year*					Total costs over 5 years	Assumptions and items costed
	*Year 1*	*Year 2*	*Year 3*	*Year 4*	*Year 5*		
McKinsey	$31–47 B	$31–47 B	$15-37B	$15-37B	$15-37B	**$**106–204 B	*Incorporates global activities including “achieve global immunization”*, *collection of complete vital statistics data*, *developing and scaling of vaccine manufacturing including universal flu vaccine development and other drug development*
WHO (low & middle income countries)	$30·8 B	$16·2 B	$16·2 B	$16·2 B	$16·2 B	$95·6 B	*Incorporate policy and coordination*, *taxes and subsidies*, *regulations and legislation*, *information collection and research*, *communication and population services*.
IHR Costing Tool	$33 B	$20 B	$22 B	*$*24 B	$24 B	*$*124 B	*Incorporate policy and coordination*, *regulations and legislation*, *information collection and research*, *veterinary services*, *biosafety and biosecurity*, *and communication and population services*.

## Discussion

Independently conducted analyses have estimated a cost of $96–204 billion USD to build country-level health capacity, globally, over a period of five years, with additional cost requirements for investments needed for regional and global coordination. These findings suggest that despite differences in underlying assumptions and approach, there is general alignment on order of magnitude cost estimates for building country-level capacity, globally. Moreover, sources are in agreement that costs are most substantial in the early stages of capacity building, highlighting the importance of early and rapid financing availability. Analyses also consistently identify personnel costs as a significant driver of overall cost requirements.

Cost estimation is complex and relies on a number of assumptions that impact results, these limitations and assumptions should be taken into account when interpreting findings. Prior research has found that countries tend to overestimate capacity based on self-report (e.g., SPAR), as compared to assessments on external evaluations (e.g., the JEE) [9]. As this analysis relies primarily on self-reported SPAR data, cost estimations may be under-estimates as compared to those conducted based on recent data for external assessments. Cost estimates also do not take into account the cost to progress from demonstrated capacity (4) to sustainable capacity (5), so countries reporting high capacity across indicators may be under-represented in cost estimates. Uncertainty in cost estimation is also influenced by factors including changes in prices of capital goods (e.g., due to supply chain disruption), inflation, and country-level decisions regarding the implementation details of specific IHR guidance (e.g., whether to rely on consultants vs. salaried employees, the specific laboratory equipment and supplies purchased, etc). Results are intended to serve as order of magnitude estimates of the global costs for country-level capacity building, not as specific estimates of individual country-level resource needs.

Moreover, this analysis relies on the first edition JEE, which has since been revised by WHO, though the most recent updated versions have not yet been officially released. The first JEE does not fully account for several key technical areas including water, sanitation, and hygiene (WASH) activities, robust infection prevention and control (IPC) programs, and support for in community health workers; these areas are likely to be significant cost drivers in future implementation efforts and are not accounted for in these cost estimates.

Reliable cost estimates also depend on clear and consistent benchmarks, particularly as they relate to known cost drivers such as recurring personnel salaries or significant capital investments. Additional clarity on workforce benchmarks (e.g., number of personnel needed per capita) will be critical to inform future planning and costing exercises, including estimates related to animal health workers, food safety workers, and skilled public health workers needed to support minimum operations necessary under the IHR. As personnel costs are known cost drivers, limited specificity in personnel-related benchmarks in the JEE may contribute to significant differences in the development of both cost estimates and national action plans; such benchmarks should be selected in an evidence-based way based on additional research on personnel needs at the national and subnational levels.

Additional financing for preparedness and response is critical for both ongoing COVID-19 response and to prepare for the next pandemic. Decision-makers tasked with developing new mechanisms and strategies for financing health security should take into account known systems-level gaps, including gaps in the staffing and laboratory capacity at the national and subnational levels. Any investment strategy in global health security must account for financing for *personnel–*identifying mechanisms to financially support the training, development, and ongoing salary requirements for those workers whose efforts underlie critical public health systems. In addition to engaging directly with impacted communities to better understand what is needed and which needs should be prioritized, structured approaches to gap identification and cost estimation such as those described here can help to ensure that investment decisions are informed by existing need, and that resources are allocated in a timely manner to the communities that need them most.

## Supporting information

S1 Table Alignment between first edition JEE, SPAR, and WHO Global Health Observatory data. (DOCX)
