# Supplementary material for: Investing in global health security: Estimating cost requirements for country-level capacity building
Source: PLOS Glob Public Health. 2022 Dec 5;2(12):e0000880. doi: 10.1371/journal.pgph.0000880 (PMC10021513; doi:10.1371/journal.pgph.0000880)
Supplement: S1 Table — (DOCX) [file pgph.0000880.s001.docx]

**Supplemental Table 1: Crosswalk of JEE scores with SPAR and WHO Global Health Observatory Data**

| **JEE 1.0** | | **SPAR** | |
| --- | --- | --- | --- |
| **Core capacity** | **Indicator** | **Core capacity** | **SPAR Indicator(s)** |
| National Legislation, Policy and Financing | P.1.1 Legislation, laws, regulations, administrative requirements, policies or other government instruments in place are sufficient for implementation of IHR. | Legislation and Financing | C.1.1 Legislation, laws, regulations, administrative requirements, policies or other government instruments to implement the IHR (2005) |
|  | P.1.2 The state can demonstrate that it has adjusted and aligned its domestic legislation, policies and administrative arrangements to enable compliance with the IHR (2005) | Legislation and Financing | C.1.1 Legislation, laws, regulations, administrative requirements, policies or other government instruments to implement the IHR (2005) |
| IHR Coordination, Communication and Advocacy | P.2.1 A functional mechanism is established for the coordination and integration of relevant sectors in the implementation of IHR. | IHR Coordination and NFP Functions | C.2.1 NFP Functions under the IHR |
|  |  | IHR Coordination and NFP Functions | C.2.2 Multi-sectoral IHR Coordination Mechanism |
| Antimicrobial Resistance (AMR) | P.3.1: Antimicrobial resistance (AMR) detection | Surveillance | C.6.1 Early Warning Function: Indicator and Event-based Surveillance |
|  |  | Health Services Provision | C 9.2 Capacity for Infection Prevention and Control (IPC) and Chemical and Radiation Decontamination |
|  | P.3.2: Surveillance of infections caused by AMR pathogens | Surveillance | C.6.1 Early Warning Function: Indicator and Event-based Surveillance |
|  |  | Health Services Provision | C 9.2 Capacity for Infection Prevention and Control (IPC) and Chemical and Radiation Decontamination |
|  | P.3.3: Healthcare associated infection (HCAI) prevention and control programs | Health Services Provision | C 9.2 Capacity for Infection Prevention and Control (IPC) and Chemical and Radiation Decontamination |
|  | P.3.4: Antimicrobial stewardship activities | Health Services Provision | C 9.2 Capacity for Infection Prevention and Control (IPC) and Chemical and Radiation Decontamination |
| Zoonotic Disease | P.4.1: Surveillance systems in place for priority zoonotic diseases/pathogens | Zoonotic Events and the Human-Animal Interface | C.3.1 Collaborative Effort on Activities to Address Zoonoses |
|  | P.4.2: Veterinary or Animal Health Workforce | Zoonotic Events and the Human-Animal Interface | C.3.1 Collaborative Effort on Activities to Address Zoonoses |
|  | P.4.3: Mechanisms for responding to infectious zoonoses and potential zoonoses are established and functional | Zoonotic Events and the Human-Animal Interface | C.3.1 Collaborative Effort on Activities to Address Zoonoses |
| Food Safety | P.5.1: Mechanisms are established and functioning for detecting and responding to foodborne disease and food contamination. | Food Safety | C.4.1 A Multisectoral Collaboration Mechanism for Food Safety Events |
| Biosafety and Biosecurity | P.6.1: Whole-of-government biosafety and biosecurity system is in place for human, animal, and agriculture facilities | Laboratory | C.5.2 Implementation of a Laboratory Biosafety and Biosecurity Regime |
|  | P.6.2: Biosafety and biosecurity training and practices | Laboratory | C.5.2 Implementation of a Laboratory Biosafety and Biosecurity Regime |
| Immunization | P.7.1: Vaccine coverage (measles) as part of a national program | Calculated based on WHO Global Health Observatory Measles Data from 2019* | N/A |
|  | P.7.2: National vaccine access and delivery | Calculated based on WHO Global Health Observatory Measles Data from 2019* | N/A |
| National Laboratory System | D.1.1: Laboratory testing for detection of priority disease | Laboratory | C.5.3 Access to Laboratory Testing Capacity for Priority Diseases |
|  | D.1.2: Specimen referral and transport system | Laboratory | C 5.1 Specimen Referral and Transport System |
|  | D.1.3: Effective modern point of care and laboratory based diagnostics | Laboratory | C.5.3 Access to Laboratory Testing Capacity for Priority Diseases |
|  | D.1.4: Laboratory quality system | Laboratory | C.5.2 Implementation of a Laboratory Biosafety and Biosecurity Regime |
| Real-time Surveillance | D.2.1: Indicator and event based surveillance systems | Surveillance | C.6.1 Early Warning Function: Indicator and Event-based Surveillance |
|  | D.2.2: Interoperable, interconnected, electronic real-time reporting system | Surveillance | C.6.2 Mechanism for Event Management (Verification, Risk Assessment Analysis, Investigation) |
|  | D.2.3: Analysis of surveillance data | Surveillance | C.6.2 Mechanism for Event Management (Verification, Risk Assessment Analysis, Investigation) |
|  | D.2.4: Syndromic surveillance systems | Surveillance | C.6.1 Early Warning Function: Indicator and Event-based Surveillance |
| Reporting | D.3.1: System for efficient reporting to WHO, FAO and OIE | IHR Coordination and NFP Functions | C.2.1 NFP Functions under the IHR |
|  | D.3.2: Reporting network and protocols in country | Surveillance | C.6.2 Mechanism for Event Management (Verification, Risk Assessment Analysis, Investigation) |
| Workforce Development | D.4.1: Human resources are available to implement IHR core capacity requirements | Human Resources | C.7.1 Human Resources to Implement IHR Capacities |
|  | D.4.2: Applied epidemiology training program in place such as FETP | Human Resources | C.7.1 Human Resources to Implement IHR Capacities |
|  | D.4.3: Workforce strategy | Human Resources | C.7.1 Human Resources to Implement IHR Capacities |
| Preparedness | R.1.1: Multi-hazard national public health emergency preparedness and response plan is developed and implemented | National Health Emergency Framework | C 8.1 Planning for Emergency Preparedness and Response Mechanism |
|  | R.1.2: Priority public health risks and resources are mapped and utilized. | National Health Emergency Framework | C 8.1 Planning for Emergency Preparedness and Response Mechanism |
| Emergency Response Operations | R.2.1: Capacity to Activate Emergency Operations | National Health Emergency Framework | C 8.2 Management of Health Emergency Response Operation |
|  | R.2.2: Emergency Operations Centre Operating Procedures and Plans | National Health Emergency Framework | C 8.2 Management of Health Emergency Response Operation |
|  | R.2.3: Emergency Operations Program | National Health Emergency Framework | C 8.3 Emergency Resource Mobilization |
|  | R.2.4 Case management procedures are implemented for IHR relevant hazards. | Health Services Provision | C.9.1 Case Management Capacity for IHR Relevant Hazards |
| Linking Public Health and Security Authorities | R.3.1: Public Health and Security Authorities, (e.g. Law Enforcement, Border Control, Customs) are linked during a suspect or confirmed biological event | IHR Coordination and NFP Functions | C.2.2 Multi-sectoral IHR Coordination Mechanism |
| Medical Countermeasures and Personnel Deployment | R.4.1 System is in place for sending and receiving medical countermeasures during a public health emergency | Health Services Provision | C.8.3 Emergency Resource Mobilization |
|  | R.4.2 System is in place for sending and receiving health personnel during a public health emergency | Health Services Provision | C.8.3 Emergency Resource Mobilization |
| Risk communication | R.5.1 Risk Communication Systems (plans, mechanisms, etc.) | Risk Communication | C.10.1 Capacity for Emergency Risk Communications |
|  | R.5.2 Internal and Partner Communication and Coordination | Risk Communication | C.10.1 Capacity for Emergency Risk Communications |
|  | R.5.3 Public Communication | Risk Communication | C.10.1 Capacity for Emergency Risk Communications |
|  | R.5.4 Communication Engagement with Affected Communities | Risk Communication | C.10.1 Capacity for Emergency Risk Communications |
|  | R.5.5 Dynamic Listening and Rumour Management | Risk Communication | C.10.1 Capacity for Emergency Risk Communications |
| Points of Entry (PoE) | POE.1 Routine capacities are established at PoE. | Points of Entry | C.11.1 Core capacity Requirements at all times for Designated Airports, Ports, and Ground Crossings |
|  | POE.2 Effective Public Health Response at Points of Entry | Points of Entry | C.11.2 Effective Public Health Response and Points of Entry |
| Chemical Events | CE.1 Mechanisms are established and functioning for detecting and responding to chemical events or emergencies. | Chemical Events | C.12.1 Resources for Detection and Alert |
|  | CE.2 Enabling environment is in place for management of chemical Events | Chemical Events | C.12.1 Resources for Detection and Alert |
| Radiation Emergencies | RE.1 Mechanisms are established and functioning for detecting and responding to radiological and nuclear emergencies. | Radiation Emergencies | C.13.1 Capacity and Resources |
|  | RE.2 Enabling environment is in place for management of Radiation Emergencies | Radiation Emergencies | C.13.1 Capacity and Resources |

Supplemental Table 1. Alignment between first edition JEE, SPAR, and WHO Global Health Observatory data. In cases where multiple SPAR indicators are listed for a single JEE indicator, the average of reported scores is taken.

* As outlined in P.7.1 of the JEE, States for which 50% of fewer of the country’s 12-month-old population has received at least one dose of measles containing vaccine are considered to have a score of 1 (no capacity); 50-69% vaccination is considered a score of 2 (limited capacity), 70-89% vaccination is considered a score of 3 (developed capacity); 90-94% vaccination is considered a score of 4 (demonstrated capacity), and 95% vaccination or higher is considered a score of 5 (sustainable capacity). Vaccination data were not available for the Holy See; however, due to assumed low infant population in Holy See, no additional measles vaccinations for infants were costed. Vaccination data for Liechtenstein were not reported; though coverage was assumed to be 95% based on average vaccination coverage among regional neighbors including Germany, Switzerland, Italy, and Austria.
